# Low-Power Low-Cost Wireless Flood Sensor for Smart Home Systems

**DOI:** 10.3390/s18113817

**Published:** 2018-11-07

**Authors:** Pedro Teixidó, Juan Antonio Gómez-Galán, Fernando Gómez-Bravo, Trinidad Sánchez-Rodríguez, Javier Alcina, Juan Aponte

**Affiliations:** 1 OnTech Security LLC, C/Hispano Aviación, 7–9, 41300 La Rinconada, Spain; pedro.teixido@ontech.es (P.T.); javier.alcina@ontech.es (J.A.); juan.aponte@ontech.es (J.A.); 2 Department Electronic Engineering, Computers, and Automatic, University of Huelva, Avda de las Artes, s/n, 21007 Huelva, Spain; fernando.gomez@diesia.uhu.es (F.G.-B.); trinidad.sanchez@diesia.uhu.es (T.S.-R.)

**Keywords:** smart homes, flood detection, wireless sensor network, low power consumption, IEEE 802.15.4

## Abstract

This paper presents the design of a wireless flood sensor to detect the presence of water on home floors, providing early warning of water leaks. A wireless sensor network has been deployed to gather the measurements from the sensor nodes. A control central coordinates the network and processes the data. Users can remotely inquire for the presence of water, status of the batteries for a specific node, the type of liquid and information about its functionality and alarms, thanks to a proprietary software application. The alerts are also communicated to the user within the home through an audible siren. The designed device is optimized in terms of costs, ease of deployment and maintenance, thus making it widely acceptable to end users.

## 1. Introduction

In recent years, many of the new smart home systems have added water leak detectors to their roster of connected devices [1,2]. These sensors can be placed near appliances, such as washing machines, dishwashers, or water heaters, or they can be mounted in basements, kitchens, bathrooms, toilets, or garages to prevent burst pipe disasters. The importance of early detection is crucial to avoid these situations.

Most smart home water sensors are battery powered and include some variety of low power wireless technology. Wireless communication technologies have advanced rapidly and the use of wireless sensor networks (WSNs) has extended to a large number of applications, such as home automation, environmental monitoring, health monitoring, etc. due to their very promising advantages [3,4,5,6,7,8,9,10]. WSN eliminates the need of wired sensor nodes across the home and reduces installation and maintenance costs. The IEEE 802.15.4 standard is particularly suited for the implementation of low cost, low power consumption, reliable control and real-time monitoring applications within the smart home framework [11,12,13]. Efficient power management is a major concern in wireless sensor nodes [14,15,16].

The implementation of the wireless device implies the integration of different subjects, such as electronics, computer science, and communication systems. The system collects information from sensors, processes the information, and activates outputs.

The proposed sensor is also an initiative for telecare and electronic assistive technology, which can help people who have difficulties, due to age or disability, to live safely and independently in their own homes [17,18,19,20,21,22].

Nevertheless, until now, there were two significant drawbacks for deploying smart sensors along isolated areas: the cost of the components, and the battery lifetime. They required periodical sensor supervision for node status check and battery replacement. As a consequence, the authors were interested in designing an integrated solution that allows long periods between maintenance and replacement visits. For this propose, this work pays special attention on achieving a new electronic design with a novel operational control mode and an efficient communication strategy, resulting in a long-term autonomy sensor with auto-checking capabilities.

The developed device is a low cost and low power consumption system that makes possible an easy integration of sensor nodes into smart home systems. The user can have remote access to the sensor information through communication networks such as Internet or mobile networks.

A competitive advantage of the proposed sensor is that the sensing electrodes detect the presence of water at two heights, so it is able to discriminate between the presence of accidental water spillage (e.g., dropping of a glass or a bucket of water) and a real flood (broken pipe, water inlet from the outside, etc.). This is a very convenient feature in order to minimize false alarms.

Power management strategies have increased spectacularly due to the use of handheld portable devices and the intensive research in the area of wireless sensor networks, which usually employ battery-operated devices. In this work, a proper choice of low power hardware components and a customized WSN solution allow to prolong the battery lifetime, extending the autonomy of the system.

This work is aimed to contribute to the trend of deploying technologies in real environments and for long-term periods by reducing the system complexity, implementing solutions that provide tangible advantages to the everyday life of the end users. This paper presents details of the design, installation, and evaluation of an integrated WSN of flood sensors for real-time alerts using the IEEE 802.15.4 wireless technology. Although the target application is in smart homes, the system can also be used for the industry since it can detect the presence of water or other moist fluids in basements, factory floors, labs, or other locations. The paper is organized as follows: Section 2 describes the hardware architecture and the functionality of the sensing node, the actuator node and the control central. Section 3 deals with the software design and the energy management. Section 4 describes the monitoring application. Finally, some conclusions are drawn in Section 5.

## 2. Hardware Design Aspects

The flood sensor is a device designed to detect water leaks in domestic environments. The wireless sensor node was developed according to requirements related to size-efficiency, low cost, and low power dissipation. It operates just like a traditional water alarm but, on the other hand, it is connected to the home network and is able to send an alert to the user, saving costs in water damage remediation.

**Figure 1:**
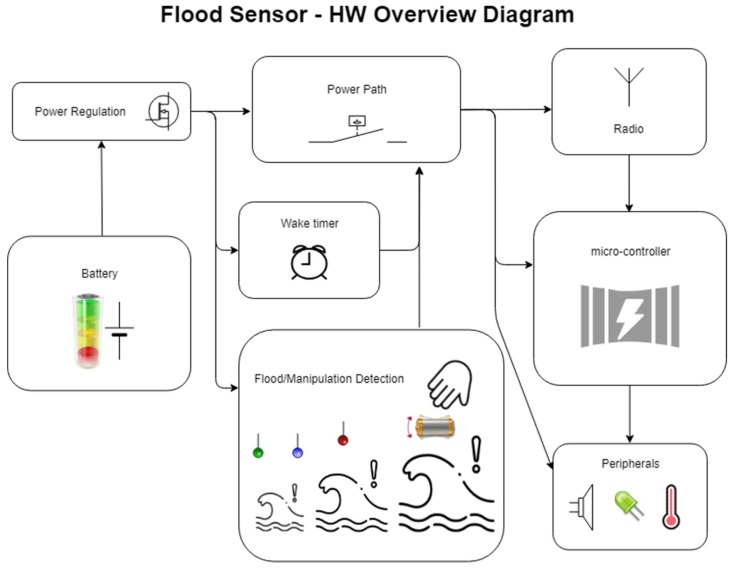
Hardware overview diagram for the flood sensor.

The deployed WSN is composed of five flood sensor nodes (although it can be easily scaled up) and a gateway or control central. A siren has been also included to provide an audible warning against water presence. The flood sensor detects the accumulation of water on a surface and data is sent through a wireless module, enabling communication with the main system. The sensor nodes can communicate with each other and with the external network via Internet or 2G/3G/4G using the control central. The sensors connect to the smart home control central and the user can inquire the device’s status by using a phone or a tablet. If the device detects water, the user gets an alert (usually a text or push notification). Moreover, the wireless flood sensing node also includes a tilt sensor (so the user knows if someone moved it), a temperature sensor, a buzzer, and a light. Figure 1 shows the overview diagram of the system.

### 2.1. Wireless Flood Sensor Node

The sensor node includes all circuitry necessary to detect the presence of water at two levels, measure the sensor’s tilt, process the data and light signals, and send alarms by radio frequency. The hardware is distributed on a single printed circuit board.

**Figure 2:**
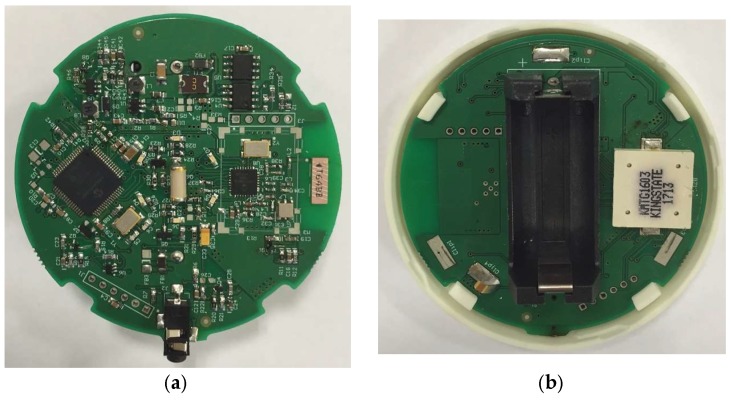
Wireless flood sensing node. (**a**) Top; (**b**) Bottom.

The flood sensor board is shown in Figure 2 and comprises: a battery; a 16-bit microcontroller for controlling the hardware, performing measurements and electronic detection of water levels; an RF (radio frequency) module; and a buzzer to warn the user about water presence. It also contains a tamper to detect the opening of the device for handling purposes or sabotage. Additionally, the sensor node includes an inclinometer, which enables the system to identify a possible water stream, and also allows knowing if the device has been relocated or manipulated. For the design of the wireless sensor node, features such as robust radio technology, low cost and low power consumption components, the use of long-lasting energy sources and reduced size have been considered.

In order to increase the lifetime of the battery, the design areas related to power management and stack protocol have been exploited. Energy consumption by node is optimized in such a way that it can be powered solely by batteries. In particular, a careful design of the flood sensor has been made in hardware design, wireless communications and firmware, so that with a single CR123 type battery it achieves an autonomy greater than 10 years.

Regarding the radio module, it transmits and receives data through a communication standard. The selection of the wireless communication protocol was determined by considering data rate, low power consumption, and cost. The IEEE 802.15.4 standard is the most appropriate to meet the requirements for the target application. It is suitable for applications where the data volume to transfer is low, and it makes possible for network nodes to be powered by battery without recharging for a long period of time. Moreover, this standard presents a high capability of network range expansion by increasing the node quantity. The radio module is based on the MRF89XA transceiver from Microchip, one of the lowest power consumption devices available, and exchanges data with the central control node via wireless communication at 868 MHz.

**Figure 3:**
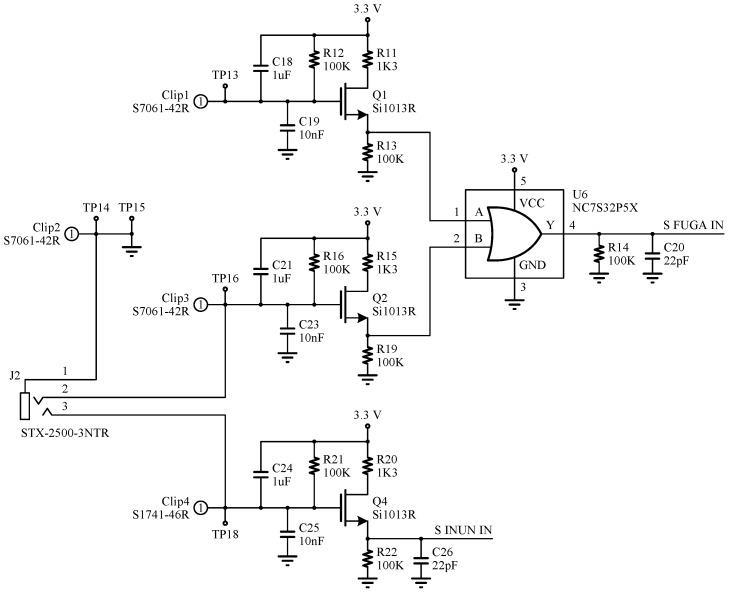
Simplified circuit schematic for the flood sensor.

Figure 3 shows the circuit schematic for the flood sensor. This circuit is responsible for measuring the two water levels, and is based on a digital circuit that detects the different liquid heights by means of electrodes placed on the bottom of the board. The circuit is designed in such a way that it has a virtually zero static current consumption, having current flow only in the presence of water, thus saving power consumption. If a flood is detected, the output of the circuit is activated and the microcontroller leaves the sleep state in which it normally stays.

**Figure 4:**
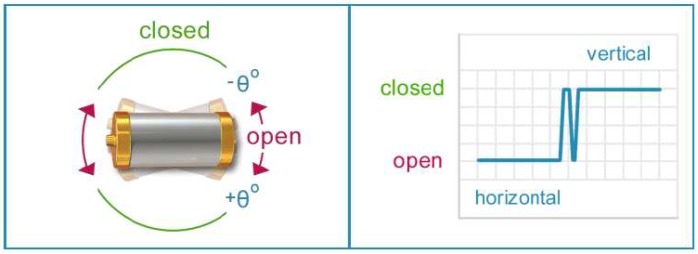
Tilt sensor capsule.

On the other hand, the tilt sensor is based on a SQ-SEN-815B capsule from Signal Quest, and it can measure inclinations of up to 15 degrees in either direction (Figure 4). As it has been mentioned above, this element is very useful for detecting movement of the sensor, either by a stream of water or flood, or by being manipulated. It is a digital circuit with practically no power consumption, so it does not compromise the autonomy and power consumption specifications pursued by this work.

**Figure 5:**
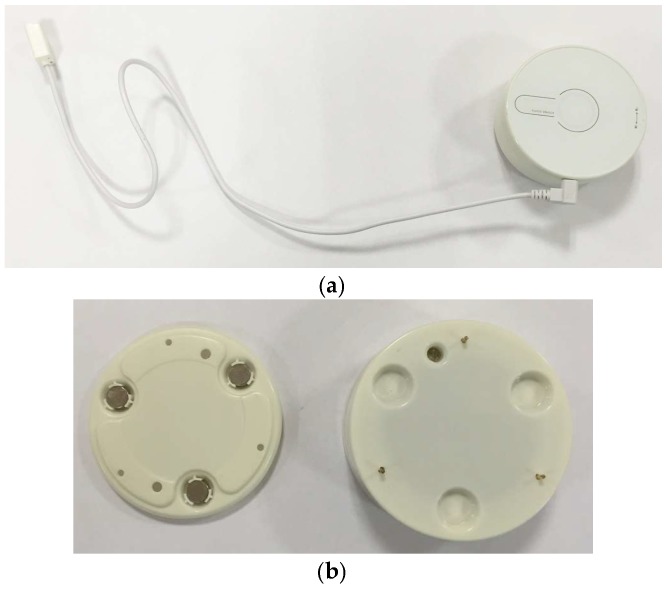
(**a**) Flood sensor packaging; (**b**) Magnetic accessory for installation on a wall.

The packaging of the flood sensor is cylindrical, as shown in Figure 5a, with electrodes that touch the flat contact surface and that detect the presence of water or other types of liquids. This sensor can be placed on the floor or on a wall. For the installation on a wall an accessory has been designed which, once fixed to the wall (with adhesive), allows the sensor to be magnetically attached to it, as shown in Figure 5b.

### 2.2. Control Central

The control central performs the data processing and the communications with the remote server, and has been designed with a modular structure. This control unit has different communication modules, both wired and wireless, and six expansion slots for general purpose modules (sensors, inputs and outputs, communications, etc.). The control system is governed by a 32-bit PIC32MZ1024 microprocessor from Microchip, with a clock frequency of up to 140 MHz, and the following characteristics: 80 I/O pins; 32 analog input channels; dedicated hardware for UART; SPI; I2C and USB communication buses; and a real-time clock (RTC). The system supports input voltages between 15 V and 36 V. The power circuit has three main modules for reducing the input voltage to 5 V, which is required to supply some digital circuits, and to obtain a dual supply voltage (±12 V) for the analog circuitry. From the battery voltage, two other voltages are generated: one of 3.3 V to supply all control parts, and a second one of 5 V for powering the communication circuits. The aim is for the control central to never lose connection to the server and be able to send alarms and events even when the mains are disconnected in the event of a power cut. A backup battery of 4500 mAh and 3.7 V was selected in order to provide enough energy to ensure connectivity for 12 h in case of emergency.

The communications have been designed in a redundant way in order to avoid losing connectivity, and thus, the control central incorporates the following types of communications: Ethernet, RS485, USB, Wi-Fi and 2G/3G/4G. Ethernet, Wi-Fi, and 2G/3G/4G communications are used to establish connectivity to a remote server or the cloud. The order of priority for network use is Ethernet, Wi-Fi, and 2G/3G/4G, so that the control central will always first connect via Ethernet. The device attempts to perform the communication by one of the other two ways if loss of connection happens, or if the user settings establish it so.

An expansion slot compatible with some industry standards, such as Xbee and click modules, has been provided for 2G/3G/4G communications. This fact increases the flexibility of the design since it allows the use of modules of different technologies and frequencies depending on the countries to which the wireless system is intended. Furthermore, for applications where the installation is local, a USB connection is included, so that the device may be connected directly to a PC. Finally, an RS485 type industrial serial communication is provided for other applications where communications between sensors, without a network connection, is needed. This interface can also be used for power supply devices connected in cascade in order to facilitate the installation. Additionally, in any of the expansion slots equipped with SPI and I2C buses, and digital and analog outputs, a communication module type ZigBee, 6LoWPAN, or similar can be connected.

All wired connections are designed with galvanic isolation by using insulator integrated circuits, in such a way that communications are isolated from the mains to prevent loss or alteration of messages in case of voltage spikes or unwanted transients.

**Figure 6:**
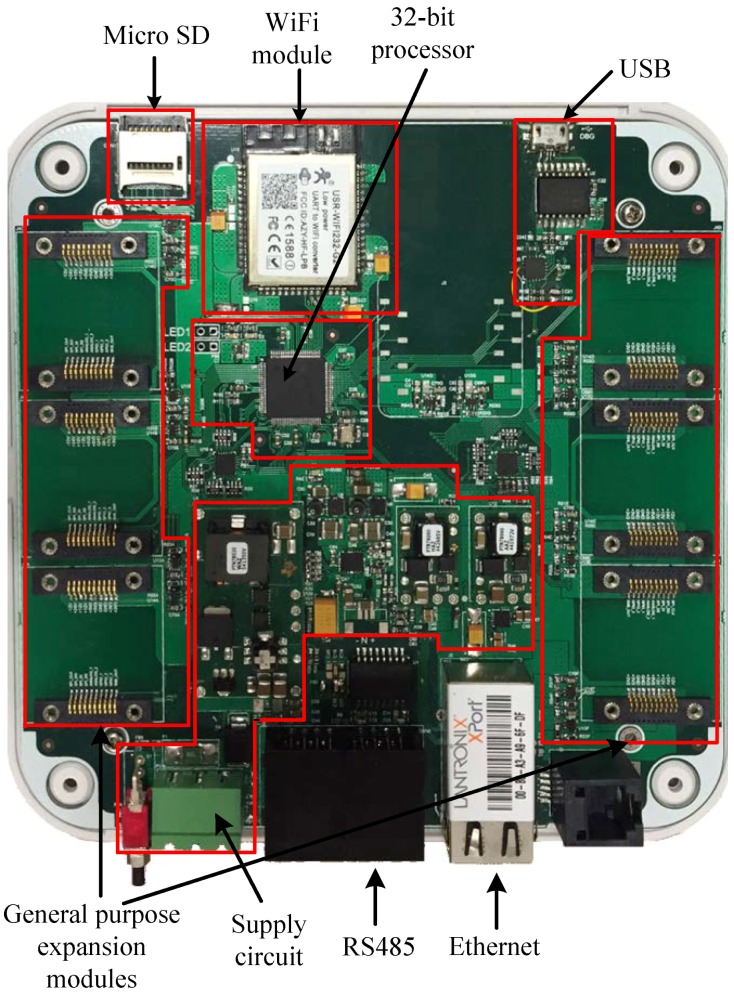
Image of the control central.

For further information reinforcement, the control central includes a storage card slot which accepts memories of up to 4 GB. The system detects that the memory is connected, and if so, will create a historical of events which can be collected by extracting the card itself, or through any of the communication interfaces when communication is restored. Each expansion module has a power control circuit which allows for the module to be disabled if necessary, putting the device into an ultra low power consumption mode and prolonging the autonomy of the internal battery. Figure 6 shows details of the hardware implementation of the control central.

### 2.3. Siren Actuator Node

The siren is the device responsible for acoustically warning users of the existence of alarms in the system, as well as to serve as a deterrent to intruders. As the other blocks of the system, the design of the siren seeks power efficiency improvements and size reduction. This device is power supplied externally, but also includes two CR123 batteries to keep it functioning in case of power failure. It can be installed on a tabletop or wall and has dimensions of 88 mm × 63 mm.

**Figure 7:**
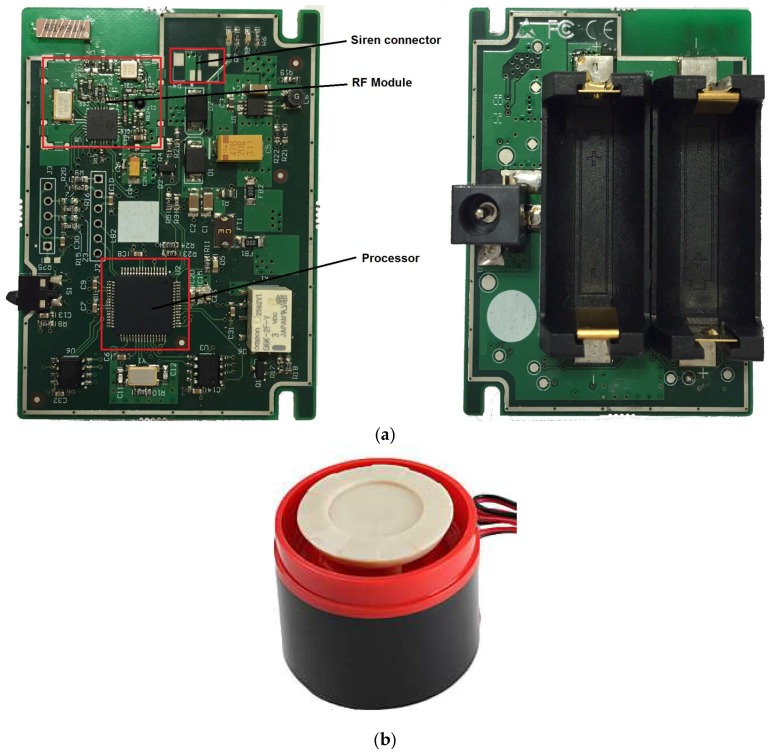
(**a**) Photographs of the siren circuitry; (**b**) Commercial siren.

The circuitry of the siren has been implemented in a single two-layer printed circuit board as shown in Figure 7a. It contains the necessary electronics for RF communications, the proper management of the siren (acoustic indication), and an anti-tampering opening mechanism. The entire electronics, which include the same 16-bit microcontroller and RF module previously described for the sensor node, are placed on the top, and the batteries and power input are place on the bottom of the PCB.

**Figure 8:**
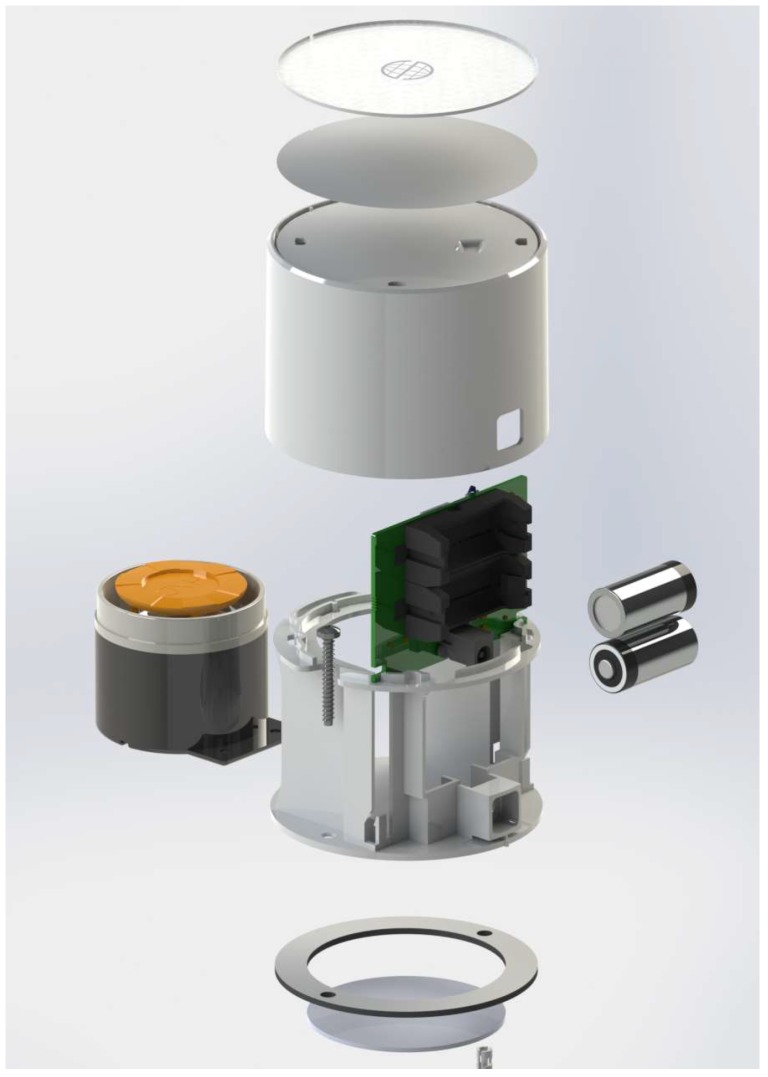
Detail of the packaging design of the siren node.

For the issuance of the acoustic signal a commercial siren of 120 dB is used, which can operate from 3 V to 12 V, and consumes a maximum power of 12 W. This siren is connected to the board with custom wires, as it is shown in Figure 7b. The packaging design of the siren actuator node is shown in Figure 8.

## 3. Power Management and Software Design

Emerging autonomous low power applications, such as miniaturized wireless sensor nodes, require efficient power management systems. The long-term operation for a wireless system requires the use of proper design strategies both at hardware and software levels. As it has been discussed previously, the hardware elements have been chosen to ensure proper functionality with low power consumption. In addition, the software also incorporates energy saving modes.

**Figure 9:**
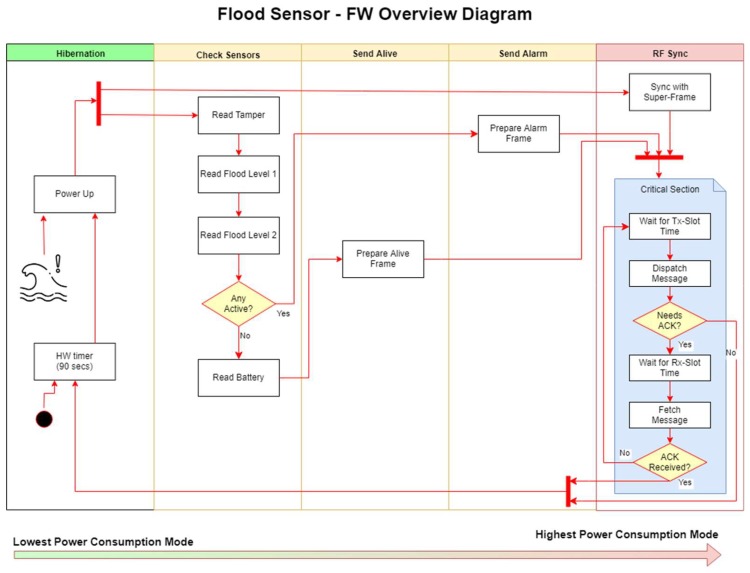
Firmware overview diagram for the flood sensor.

On the flood sensor node, ultra-low power consumption is achieved by keeping the microcontroller powered off (with no power supply) most of the time. A discrete hardware timer is in charge of periodically powering up the microcontroller to send the alive signal. In case of the flood sensor the hardware is also prepared to instantly power up the microcontroller in the event of water detection. This feature eliminates the need to periodically check the water sensor, and permits to extend the power-up interval to tens of minutes or even a few hours. Figure 9 shows a high-level view of the finite state machine implemented by the software. Every time the microcontroller is powered, the wake source needs to be analyzed. If no water alarm is found, an alive message is sent to the portal, along with the status information (battery level, RF signal strength, etc.). Once the alive message is acknowledged by the control unit, the microcontroller releases its power supply and returns back to its unpowered state. The whole operation typically takes a few milliseconds, operating with an average current of around 12 mA.

If a water alarm is found when the wake source is analyzed, the process is very similar to the alive process, with an alarm message being sent instead of an alive message. The duration of the task is the same, and the resources used are also similar. On the other hand, the likelihood of occurrence of such an event is really small, therefore this event can be ignored when estimating battery life time.

The current implementation of the flood sensor drops the duty cycle (system in active mode) down to approximately 0.001%. Such a low duty cycle makes possible to extent the device battery life up 10 years on a CR123 battery.

**Figure 10:**
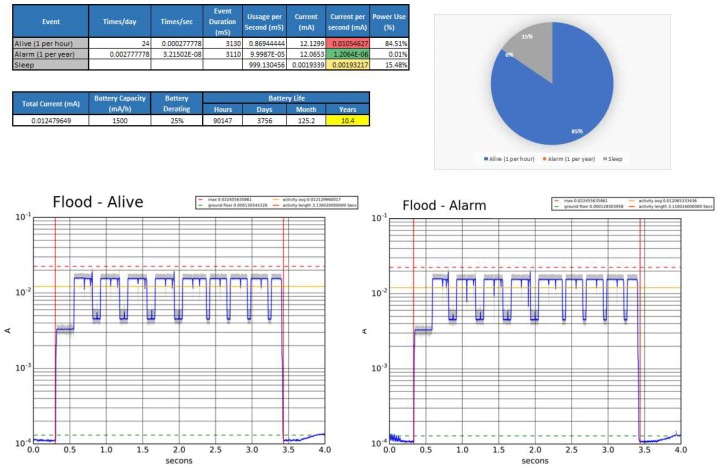
Detail estimation of the flood sensor battery life.

Figure 10 shows a detailed battery life estimation. The process consists on sampling in detail the current consumption for each of the different evens (in the case of the Flood sensor only Alive and Alarm), as well as the quiescent current consumption. Then, an excel file is created with the consumptions and the periodicities of each event type. The battery life estimation is finished by calculating the available battery capacity after a theoretical derating, divided between the equivalent DC consumption of the device. In the case of the Flood sensor, and using a 1500 mAh battery, the estimated battery life is 10.4 years.

Due to the nature of the device, the current is rapidly changing from a few µA to tens of mA, so measuring the energy drawn during one of these events becomes a challenge. The way it has been performed is with a tool that simultaneously captures the power consumption in four different ranges. This data is latter mixed by a script that selects the data from the channel which is better suited for the current flowing measured at the time of each particular sample. The script also uses a calibration file generated at the moment of the test, which minimizes the sampling error. Finally, a logarithmic plot, where the pattern of the event can be easily evaluated, is generated. The plot also includes the event length and the average current drained during the event.

This power estimation is used by development engineers to monitor what event type is draining more power, and at what stage of the event the power is being used. Such information is key to allow engineers to focus their efforts into the correct task in order to bring battery life up to specifications.

It is important to mention that the approach of powering down the system presents some challenges due to the microcontroller losing its RAM retention when powered off. That forces the firmware to store the execution context in the node’s nonvolatile memory. In order to reduce cost and failure rate it has been decided to use the internal flash memory of the microcontroller. This type of memory typically supports around 100 Kcycles, which is sufficient to safely store the execution context at the duty cycle for the expected lifetime of the device.

The control unit uses an external power supply. For this reason, the reduction of its power consumption is much less important than reducing the power consumption of the nodes. An important part of the energy spent on these nodes is associated to communications, and here is where the control unit can bring an important power saving for the associated nodes by using time slot techniques.

The control unit maintains a list of trusted nodes. It schedules the time for each of them to listen and to transmit. The control unit periodically broadcasts a frame to let all nodes know when they should listen or talk, if needed. This mechanism drops the probability of collision to nearly zero, avoiding packet retries and extending node battery life.

Channel hopping techniques have been used along with the time slot approach. The combination of both techniques is known as Time Slot Channel Hopping (TSCH). A TSCH stack not only avoids collision between system nodes, but can also handle strong radio electrical noise blocking one or more channels. The control unit shares channel hopping schemes with its associated nodes. By using and agreed scheme all nodes can predict what channel to use on each slot. Furthermore, the control unit can perform blacklisting of channels in order to maintain bandwidth when some channels are being blocked or are forbidden for any reason.

A small inconvenient of this technique is that the node enrollment becomes more complex. By definition a node cannot talk until it receives permission from a control unit. Therefore auto-discovery of nodes cannot be done unless a specific slot is put in place for such a purpose. An alternative approach to auto-discovery is to update the list of trusted nodes through other channels. In this case, the user can add nodes to the system through the website, the iOS app or the Android app. Once this is done, a remote server requests to the control unit to register a new node on its list of trusted nodes. From this point on, the control unit assigns slots to the specific node, and so they can communicate.

Once the enrolment process has been complete, the system becomes autonomous, ensuring high readability even if all communications with the remote server are lost.

## 4. Experimental Results and Monitoring Application

**Figure 11:**
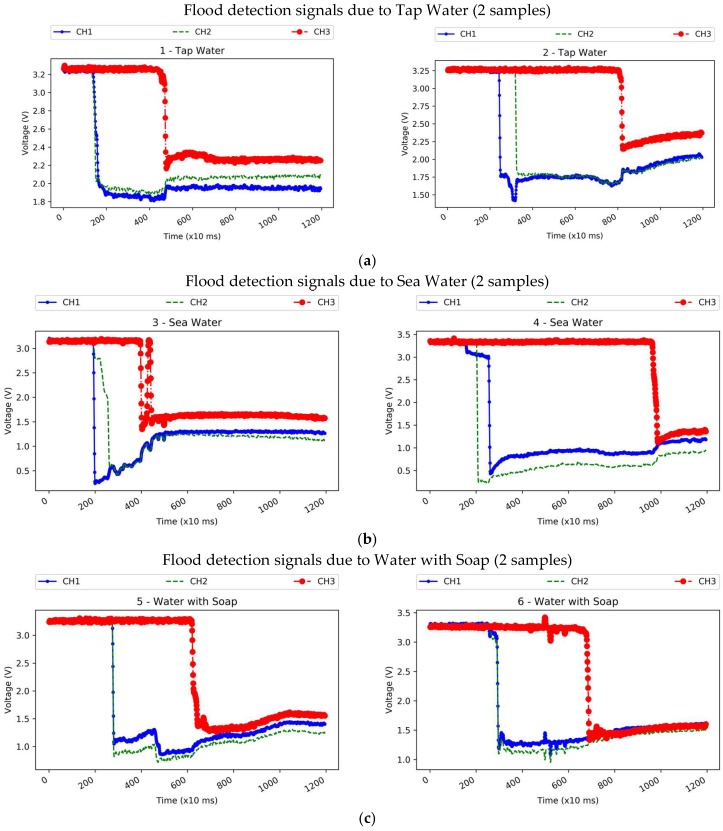
Flood detection signals. (**a**) Tap water; (**b**) Sea water; (**c**) Water with soap.

The sensor has the ability not only to detect water, but to distinguish liquids. Figure 11 shows some liquid impedance plots. The red signal corresponds to the second flooding level; therefore, it becomes excited latter on in all plots. The blue and green signals are the two electrodes at floor level. As it can be appreciated in the above plots, there are differences in the characteristic impedance of the liquids and the trends they generate during the first seconds after an activation. In case of an alarm, the value of all electrodes is sampled and sent to the cloud, where it is combined and process accordingly by one server to estimate the nature of the liquid.

**Figure 12:**
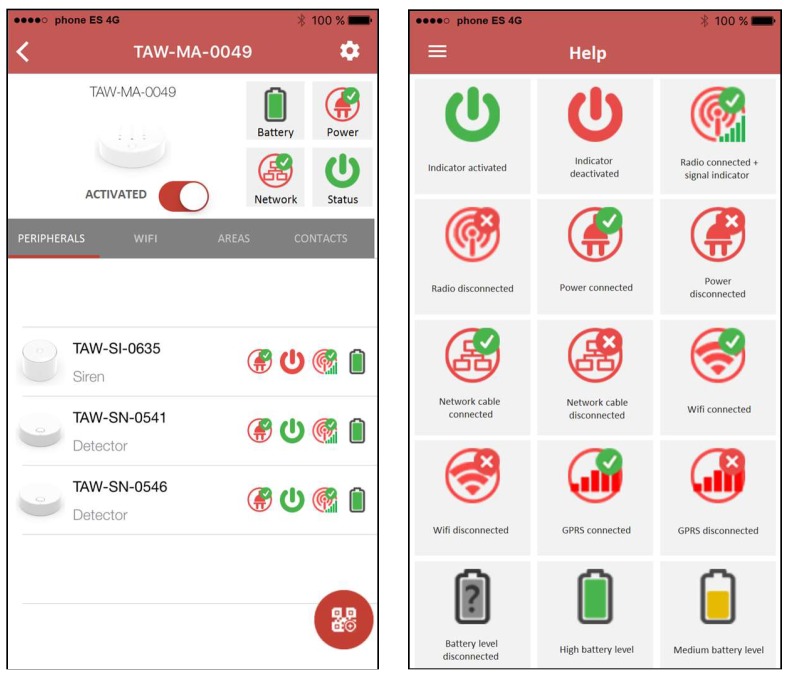
Some views of the mobile monitoring application.

The wireless system has been developed so that it can include other measurable variables inside the home, so all home systems are integrated into a centralized control unit, accessible from different wireless mobile devices, such as smartphones or tablets. Thus, the control central interacts with the end user through a web server for real-time data display, while an iOS or Android application is running on mobile terminals. Figure 12 shows some views of the mobile application used for monitoring and control, with its different functionalities.

## 5. Conclusions

A low power flood detection system for wireless sensor networks has been presented. The designed sensor allows end users to more easily monitor home devices locally and remotely, and is able to alert the user in case of home accidents. The sensor has the ability not only to detect water, but to distinguish liquids. A wireless sensor network has been deployed, where flood sensing nodes, actuator nodes and a control central have been developed. For each node, hardware and software components have been described in detail. Communication between the sensor network and the control central has been successfully implemented using the IEEE 802.15.4 standard. A graphical user interface for mobile applications allows remote access and real-time control and monitoring of several performance parameters. The result is a low cost, highly reliable and easily scalable infrastructure with application to smart homes. Although the liquid classification system is still a bit basic, it enables the possibility to machine learning algorithms expanding the number of liquids that could be distinguished, increasing the identification success rate, and it may even bring new features to the product, like home appliance preventive maintenance, by digging down activity patterns.
